# A novel Pfs38 protein complex on the surface of *Plasmodium falciparum* blood-stage merozoites

**DOI:** 10.1186/s12936-017-1716-0

**Published:** 2017-02-16

**Authors:** Gourab Paul, Arunaditya Deshmukh, Inderjeet Kaur, Sumit Rathore, Surbhi Dabral, Ashutosh Panda, Susheel Kumar Singh, Asif Mohmmed, Michael Theisen, Pawan Malhotra

**Affiliations:** 1 Malaria Group, International Centre for Genetic Engineering and Biotechnology (ICGEB), New Delhi, India; 2 Department of Clinical Biochemistry, Immunology and Genetics, Statens Serum Institute, Copenhagen, Denmark; 3 Centre for Medical Parasitology at Department of International Health, Immunology and Microbiology, University of Copenhagen, Copenhagen, Denmark; 4 Department of Infectious Diseases, Copenhagen University Hospital, Rigshospitalet, Copenhagen, Denmark; 5 Department of Biotechnology, All India Institute of Medical Sciences, New Delhi, India; 6 Department of Microbiology, All India Institute of Medical Sciences, New Delhi, India

**Keywords:** *Plasmodium falciparum*, 6-cys proteins, Glycophorin A, MSP-1_65_

## Abstract

**Background:**

The *Plasmodium* genome encodes for a number of 6-Cys proteins that contain a module of six cysteine residues forming three intramolecular disulphide bonds. These proteins have been well characterized at transmission as well as hepatic stages of the parasite life cycle. In the present study, a large complex of 6-Cys proteins: Pfs41, Pfs38 and Pfs12 and three other merozoite surface proteins: Glutamate-rich protein (GLURP), SERA5 and MSP-1 were identified on the *Plasmodium falciparum* merozoite surface.

**Methods:**

Recombinant 6-cys proteins i.e. Pfs38, Pfs12, Pfs41 as well as PfMSP-1_65_ were expressed and purified using *Escherichia coli* expression system and antibodies were raised against each of these proteins. These antibodies were used to immunoprecipitate the native proteins and their associated partners from parasite lysate. ELISA, Far western, surface plasmon resonance and glycerol density gradient fractionation were carried out to confirm the respective interactions. Furthermore, erythrocyte binding assay with 6-cys proteins were undertaken to find out their possible role in host-parasite infection and seropositivity was assessed using Indian and Liberian sera.

**Results:**

Immunoprecipitation of parasite-derived polypeptides, followed by LC–MS/MS analysis, identified a large Pfs38 complex comprising of 6-cys proteins: Pfs41, Pfs38, Pfs12 and other merozoite surface proteins: GLURP, SERA5 and MSP-1. The existence of such a complex was further corroborated by several protein–protein interaction tools, co-localization and co-sedimentation analysis. Pfs38 protein of Pfs38 complex binds to host red blood cells (RBCs) directly via glycophorin A as a receptor. Seroprevalence analysis showed that of the six antigens, prevalence varied from 40 to 99%, being generally highest for MSP-1_65_ and GLURP proteins.

**Conclusions:**

Together the data show the presence of a large Pfs38 protein-associated complex on the parasite surface which is involved in RBC binding. These results highlight the complex molecular interactions among the *P. falciparum* merozoite surface proteins and advocate the development of a multi-sub-unit malaria vaccine based on some of these protein complexes on merozoite surface.

**Electronic supplementary material:**

The online version of this article (doi:10.1186/s12936-017-1716-0) contains supplementary material, which is available to authorized users.

## Background

Malaria is a worldwide health problem, with 250 million people at risk and an estimated 438,000 deaths each year [1]. Despite introduction of effective drugs and vector control tools, elimination or even eradication of malaria remains a distant prospect. Development of an efficacious vaccine would be an important tool to combat malaria. Since morbidity and mortality is associated with asexual parasite forms [2], blood-stage antigens have been major targets for vaccine development efforts. Unfortunately, all attempts to develop a malaria vaccine based on surface proteins from the *Plasmodium falciparum* merozoite have so far failed [3–6] possibly due to insufficient understanding of the molecular architecture of the merozoite surface proteins and their organization on the merozoite surface.

Protein complexes are critical for host-pathogen interactions and for many of the biological processes involved in intercellular contacts [7]. Two merozoite surface protein complexes have a well-documented role in the invasion of erythrocytes. These are the *P. falciparum* merozoite surface protein-1 complex and the apical membrane antigen 1/rhoptry neck (RON)-complex [8–13]. A family of proteins referred to as 6-Cys domain proteins have recently gained interest as vaccine candidate antigens because of their critical role for parasite growth in the infected hepatocyte and in the mosquito midgut [14, 15]. Ten members of the 6-Cys family have been described in *Plasmodium* species that infect primates, rodents or birds [16, 17]. These proteins contain modules of six conserved cysteine residues forming three intramolecular disulfide bonds between C1–C2, C3–C6 and C4–C5. The numbers of 6-Cys modules vary from two to seven while the length of interspersed sequences between these modules varies from 7 to 160 aa [16, 18, 19]. The repeat units found in these proteins show double domain characteristics and are termed A-and B-type domains [18]. Several of the 6-Cys proteins are attached to the outer leaflet of the plasma membrane by GPI anchors, while a few are associated with the parasite surface through protein–protein interactions [17, 20].

Pbs36 and Pbs36p, the two members of 6-Cys protein family are located on the surface of *Plasmodium berghei* sporozoites [14] and knock-outs of the corresponding genes resulted in cessation of parasite development in infected hepatocytes [14, 21]. Accordingly, *P. berghei* Pbs36 and Pbs36p knock-out sporozoites failed to progress to the asexual blood stage in infected mice. Since, these mice were protected from a subsequent challenge infection with wild-type *P. berghei*, some have advocated that Pfs36 and Pfs36p knock-out parasites may be developed for an attenuated vaccine [21–23]. The two sexual-stage 6-Cys proteins, Pfs230 and Pfs48/45, are well-established candidates for a transmission blocking vaccine because of their critical role in parasite fertilization and growth in the mosquito midgut [24–28]. In comparison to the 6-Cys proteins of the hepatic and transmission stages, little is known about the 6-Cys proteins expressed during the asexual blood stages. Four members of *P. falciparum* 6-Cys family, Pfs92, Pfs41, Pfs38 and Pfs12, are expressed at the asexual blood stages. Among these proteins Pfs41 and Pfs12 form a heterodimer on the merozoite surface and Pfs92 interacts with factor H that is recruited by merozoites to evade the human complement system [20, 29, 30].

Here, the association of Pfs38, Pfs41 and Pfs12 with each other and with other merozoite surface proteins was investigated using biochemical and several protein–protein interaction tools. The existence of a Pfs38 protein complex on merozoite surface and its interaction with human red blood cells (RBCs) were also explored. The analysis of the seroreactivity of members of the Pfs38 merozoite surface complex show that these proteins are strongly recognized by naturally acquired antibodies from geographically distant areas, suggesting a functional role for this complex during the natural infection.

## Methods

### In vitro *Plasmodium falciparum* culture

*Plasmodium falciparum* strain 3D7 was cultured on human erythrocytes (4% haematocrit) in RPMI 1640 media (Invitrogen) supplemented with 10% O+human serum using standard protocol described by Trager and Jensen [31]. Parasite cultures were synchronized by two consecutive sorbitol treatments 4 h apart following the protocol described by Lambros and Vanderberg [32].

### Cloning of Pfs38, Pfs12 and Pfs41 and PfMSP-1_65_, GLURP (R2, R1, R0), SERA5

Pfs38 gene encompassing **aa 22–328** was codon optimized and synthesized from Genescript Inc, USA. The 6-Cys domain of Pfs38 (aa **153–328**) was PCR amplified from the synthetic gene using the primer pair: forward 5′ GCCCATGGGATCCAAAAAGGTGCTGCGTATTCACATCTCTAACGG 3′ and reverse 5′ GCGTCGACCTCGAGAATTTCTTCGCGTTC 3′ and cloned in pGEMT vector (Promega corporation, USA). The fragment was sub-cloned in the pET 28b expression vector between *Nco*I and *Xho*I sites. Pfs41 gene encompassing **aa 28–378** and Pfs12 gene encompassing **aa 26–323** was codon-optimized and synthesized from Genescript Inc, USA and subsequently sub-cloned between NcoI/XhoI site of pET28b.

PfMSP-1_65_ encompassing **aa 1052–1664** was amplified by PCR from *P. falciparum* 3D7 cDNA using primer pair forward.

5′ GCGCGGCCGCATCCATGGGACAGT TATCATTTGATTTATA TAATAAA 3′ and reverse 5′ GCGTCGACCTCGAGTGCATCACATCCACCATTATTTTC 3′ and cloned in pGEMT vector (Promega corporation, USA). The fragment was sub-cloned in the pET 28b expression vector between *Nco*I and *Xho*I site. GLURP (R2, R1, R0 fragments) and SERA5 was cloned as described earlier [33, 34].

### Expression and purification of recombinant proteins

The pET28b plasmids having Pfs38 and PfMsp-1_65_ genes were transformed in BL21 cells, while plasmids having Pfs12 and Pfs41 fragments were transformed in Rosetta cells. Each culture was induced at 1 mM IPTG for 5 h at 37 °C. The cells were disrupted by sonication in Tris-buffer (0.05 M Tris pH 8.0 and 0.15 M NaCl) with 9-s pulses at 9-s intervals for ten times using mini probe. The soluble and insoluble fractions were separated by centrifugation and analysed by SDS-PAGE. The expressed proteins Pfs38, Pfs12 and Pfs41were found to be present in inclusion bodies, while PfMSP-1_65_ was found to be expressed as soluble protein. To purify the recombinant proteins, each *Escherichia coli* culture was induced with 1 mM IPTG for 5 h at 37 °C. The cell pellet was suspended in 1/20 volume of lysis buffer (0.05 M Tris, pH 8.0, 0.15 M NaCl, 0.01 M DTT, 100 µg/mL lysozyme, 1 mM PMSF, 1% Triton X-100). For Pfs38, Pfs41 and Pfs12 purification, the cell suspension was sonicated and centrifuged at 12,000×*g* for 30 min at 4 °C. Pellets were washed four times with Tris-buffer without Triton X-100. The inclusion bodies thus obtained were resuspended in 8 M urea. The suspension was incubated for 30 min at room temperature and centrifuged at 12,000×*g* for 30 min at 4 °C. Supernatant containing solubilized protein was kept for binding with Ni^+2^-NTA^+^ resin for 1 h at 4 °C with constant shaking. After binding, suspension was packed in a purification column and the resin was washed four times with 8 M urea buffer containing 10 mM imidazole. Bound protein(s) was eluted in lysis buffer containing 8 M urea with different concentrations of imidazole. Eluted protein fractions were analysed on 10% SDS-PAGE. Fractions containing almost pure protein were pooled and refolded by gently diluting the protein 40-fold in refolding buffer (0.05 M Tris pH-8, 1 M Urea, 1 mM EDTA, 0.5 M arginine, 0.4 mM triton X, 1 mM reduced glutathione, 0.5 mM oxidized glutathione) with constant stirring at 4 °C for 24 h. The refolded proteins were concentrated and dialyzed against 0.05 M Tris pH-8, 0.15 M NaCl and stored at −80 °C.

For the purification of PfMSP-1_65_, the cell suspension was sonicated and centrifuged at 12,000×*g* for 30 min at 4 °C and the supernatant was incubated with Ni^+2^-NTA^+^ resin for 1 h at 4 °C. After binding, suspension was packed in a purification column and the resin was washed four times with non-denaturing buffer, 0.05 M Tris pH-8, 0.3 M NaCl containing 10 mM imidazole. Bound protein(s) was eluted in 0.05 M Tris pH-8, 0.15 M NaCl buffer containing different concentrations of imidazole. Eluted protein fractions were analysed on 10% SDS-PAGE. Fractions containing almost pure proteins were pooled and dialyzed against 0.05 M Tris pH-8, 0.15 M NaCl. The expressed proteins were run in reduced and non-reduced conditions and confirmed by Western blot analysis with monoclonal Anti-His antibody (Sigma Aldrich, USA). GLURP (R2, R1, R0 fragments) and SERA5 were expressed and purified as described earlier [33, 34].

### Generation of antibodies against Pfs38, Pfs12 and Pfs41, PfMSP-1_65_, GLURP (R0, R1 and R2 fragment) and SERA-5

Antibodies against Pfs38, Pfs12 and Pfs41 and PfMSP-1_65_ were raised in both mice and rabbit. The animals were housed and handled in accordance with the institutional and national guidelines. The Institutional Animal Ethical Committee at ICGEB, New Delhi, India, approved the animal use protocol described in the studies. The animals were bred under the guidelines of the authorizing committee. For the antibody generation, 5–6 weeks old female BALB/c mice were immunized with 25 μg of each protein: rPfs38, rPfs12 and rPfs41 and rPfMSP-1_65_ emulsified in Freund’s complete adjuvant on day 0, followed by three boosts of proteins emulsified with Freund’s incomplete adjuvant on days 14, 28 and 42. The animals were bled for serum collection on day 49. For rabbit immunization, New Zealand white female rabbits were immunized with 200 μg of either of the following recombinant proteins: rPfs38, rPfs12 and rPfs41 and rPfMSP-1_65_ emulsified in Freund’s complete adjuvant on day 0, followed by three boosts emulsified with Freund’s incomplete adjuvant on days 21, 42 and 63. The animals were bled for serum collection on day 70. Antibody titres in serum samples were quantified by enzyme-linked immunosorbent assay (ELISA). Antibodies against GLURP (R2, R1 and R0 fragments) and SERA5 were raised according to protocols described earlier [33, 34].

### Immunoblot analysis of *Plasmodium falciparum* merozoites

Briefly, *P. falciparum* merozoites were harvested and lysed with SDS sample buffer and boiled at 98 °C for 15 min. After removing insoluble material by centrifugation (15,000×*g* for 15 min), the parasite lysate was run on SDS-PAGE and transferred to nitrocellulose membrane. The membranes were probed with rabbit anti-Pfs38 (1:1000) or anti-Pfs12 (1:1000) or anti-Pfs41(1:1000) antisera followed by goat anti-rabbit HRP conjugated secondary antibody (1:3000). The membranes were developed with DAB/H_2_O_2_ solution (Sigma Aldrich, USA).

### Immunoprecipitation

Parasites in late schizont stage (44–46 hpi) were collected by saponin lysis and washed several times to remove RBC contamination. Lysates for immunoprecipitation were prepared in IP lysis buffer (250 mM Tris, 150 mM NaCl, 1 mMEDTA, 1% NP-40, 5% glycerol; pH 7.4). Protein-A Agarose were used to bind 20 µg of polyclonal anti-Pfs38 antibody or anti-PfMSP-1_65_ antibody or anti-Pfs 12 or anti-Pfs41 antibody while Protein-A Agarose incubated with 20 µg of pre-immune antibody served as a control. After 12 h of incubation at 4 °C, each antibody bound protein-A Agarose was washed with PBS and incubated with parasite lysate (~1 mg per 10 μL of Agarose). After 12 h of incubation, protein-A Agarose beads were washed with wash buffer and bound proteins were eluted from the beads using the elution Buffer (Tris–Glycine pH 2.8). Proteins in the immunoprecipitated samples were digested by in-solution trypsin digestion method. Samples were brought to a final volume of 100 µL in 50 mM ammonium bicarbonate (Sigma) buffer to adjust the pH to 7.8, reduced with 10 mM DTT (final concentration) for 1 h at room temperature (RT) and alkylated with 40 mM iodoacetamide (Sigma Aldrich, USA) for 1 h at RT under dark conditions. Proteins were digested by the addition of Promega sequencing grade modified trypsin (V511A) at a ratio 1:50 (w/w) of trypsin:protein. For complete digestion, samples were placed in a water bath at 37 °C for 18 h. After digestion, extracted peptides were acidified to 0.1% formic acid and analysed by Orbitrap VelosPro mass spectrometer coupled with nano-LC 1000 (Thermofisher Scientific Inc, USA).

### ELISA

In vitro interaction between members of the Pfs38 complex was examined by ELISA, as described previously with minor modifications [35]. Briefly, 96-well microtitre plates were coated overnight with 2 µg/mL of Pfs38 at 4 °C. After blocking the wells with 5% skimmed milk in PBS, recombinant GLURP R0, GLURP R1, GLURP R2, PfMSP-1_65_, PfMSP-1_19_, Pfs41, Pfs12 and SERA5 were added in increasing concentrations of 2, 5, 10, and 20 µg/ml and plates were incubated for 2 h. For the ELISA interaction of glycophorin A with Pfs38, 1 µg/mL of glycophorin A was coated in microtitre plates at 4 °C and after blocking, increasing concentrations of Pfs38 or GLURP R2 or PfMLH (non-specific protein) were added and plates were incubated for 2 h. For the inhibition assay, 2 µg/mL of Pfs38 was coated at 4 °C and after blocking, anti-Pfs38 antibodies were added in increasing concentration of 0.25, 0.5, 1 and 2 mg/mL and incubated for 30 mins. This was followed by incubation with glycophorin A protein at 20 µg/mL for 2 h. Bound proteins were detected through polyclonal antibody raised against the respective proteins for 1 h. Horseradish peroxidase (HRP)-conjugated anti-mouse or anti-rabbit antibodies (1:3000) were added in each well for 1 h and binding was quantified after adding the substrate of phenylenediaminedihydrochloride (Sigma Aldrich, USA) by measuring the resulting absorbance at 490 nm in an ELISA microplate reader. All the experiments were done in triplicate and mean ± SEM was calculated.

### Far western

Far western assay was carried out according to the protocol described earlier [36]. Briefly 1–5 µg of recombinant Pfs38, Pfs41, Pfs12, PfMSP-1_65_, PfMSP-1_19_, and a non-specific protein PfMLH were separated by SDS-PAGE and transferred to a membrane. The proteins on the membranes were denatured and renatured. These membranes were blocked with 5% skimmed milk and incubated with 2 µg/mL of purified interacting bait proteins, i.e., recombinant GLURP R2 or Pfs38 or SERA-5 in protein-binding buffer (100 mM NaCl, 20 mM Tris (pH 7.6), 0.5 mM EDTA, 10% glycerol, and 1 mM DTT) for 2 h at RT. Membranes were washed to remove the non-specific interactions and were incubated with rabbit anti-GLURP R2 (1:1000) or rabbit anti-Pfs38 (1:1000) or mouse anti-SERA5 antisera (1:500) overnight at 4 °C followed by incubation with goat anti rabbit IRDye 800CW (1:15,000) or goat anti mouse IRDye 800CW (1: 15,000) for 2 h at RT. Finally, the membranes were imaged with a LI-COR Odyssey FC instrument.

### Surface plasmon resonance (SPR) analysis

SPR assays were performed on BIACORE 2000 instrument (GE Healthcare) at 37 °C, using HBS-EP buffer (general purpose buffer, degassed and ready to use 0.01 M HEPES pH 7.4, 0.15 M NaCl, 3 mM EDTA, 0.005% v/v Surfactant P20; GE Healthcare Lifesciences, USA). Recombinant GLURP R2 was immobilized up to 400 response units in a flow cell of CM5 sensor-chip (GE Healthcare Lifesciences, USA). For kinetic measurements, increasing concentrations of recombinant Pfs38 were injected over immobilized GLURP R2 as well as the reference flow cell, at a flow rate of 20 μL/min. The surfaces were regenerated with a pulse of 10 mM Glycine at pH 1.5 at the end of each injection cycle. Duplicate injections of the same concentration in each experiment were superimposable, demonstrating no loss of activity after surface regeneration. Reference-subtracted sensorgrams were analysed using the Biacore evaluation software 4.1.1 (GE Healthcare Lifescienes, USA). To determine the kinetic parameters of the interaction, binding responses in the steady-state region of the sensorgrams were plotted against analyte concentration and fitted to the standard 1:1 (Langmuir) bimolecular interaction with simultaneous fitting of ka and kd.

### Glycerol density gradient centrifugation

Briefly, schizont-stage parasite were lysed in 0.5% Nonidet P-40/Hepes-buffered saline (10 mM Hepes at pH 7.0, 150 mM NaCl, 2 mM MgCl_2_, 10 mM KCl, 0.5 mM EDTA, and protease inhibitors). Lysate was cleared by centrifugation and 500 μL of lysate was layered on top of a 9-mL 5–45% step glycerol gradient. Gradients were centrifuged at 38,000 rpm for 18 h at 4 °C ina SW41 rotor (Beckman). Twenty fractions of 0.5 mL each were collected from each gradient and equal volumes of each fraction were mixed with loading dye. Protein samples were resolved by SDS/PAGE and analysed by Western blotting using anti-Pfs38, anti-Pfs12, anti-Pfs41, anti-GLURP R2, anti-PfMSP-1_65_ and anti-SERA5 antibody. In-solution trypsin digestion of fraction 5 followed by LC–MS/MS analysis was done to confirm the protein.

### Indirect immunofluorescence assay (IFA)

Confocal laser scanning IFAs were performed with *P. falciparum* asexual blood stages. Cell fixation, antibody incubation and imaging were performed by standard techniques and microscopic examination was performed using a A1 confocal microscope (Nikon Instruments Inc, USA). Images were analysed using Nikon NIS Elements v 4.0 software. Imaris image was created using the software IMARIS v 4.0.

### Erythrocyte binding assay

Erythrocyte binding assays were carried out as described previously [37]. Neuraminidase treatment of RBC was carried out as described previously [37].

### Invasion inhibition assay

Anti-Pfs38 IgG and pre-immune IgG were added to highly synchronized schizont-stage cultures (2% haematocrit and 1% parasitaemia) at final concentration of 2, 5 and 10 mg/mL. The cultures were incubated for 40 h for schizont rupture and merozoite invasion. Parasitaemia was counted by flow cytometry. Percentage inhibition was calculated relative to the pre-immune sera. Bars indicate mean ± SEM of duplicate measurements.

### Seroreactivity analysis

ELISA analysis was performed to determine the seroreactivity of the proteins of the Pfs38 complex using sera from naturally infected malaria patients as described earlier [38]. Sera from 28 *P. falciparum* malaria patients in India and 28 *P. falciparum* malaria patients in Liberia was used, while sera from 28 Danish volunteers was used as a negative control. For assessing the conformation of three refolded 6-cys proteins; i.e. Pfs38, Pfs12 and Pfs41 as well as PfMsp-1_65_ these proteins were denatured with 8 M Urea and seroreactivity was tested using 28 Liberian sera samples.

## Results

### Expression and purification of recombinant Pfs41, Pfs38, Pfs12 and PfMSP-1_65_ proteins in *Escherichia coli*

**Fig. 1:**
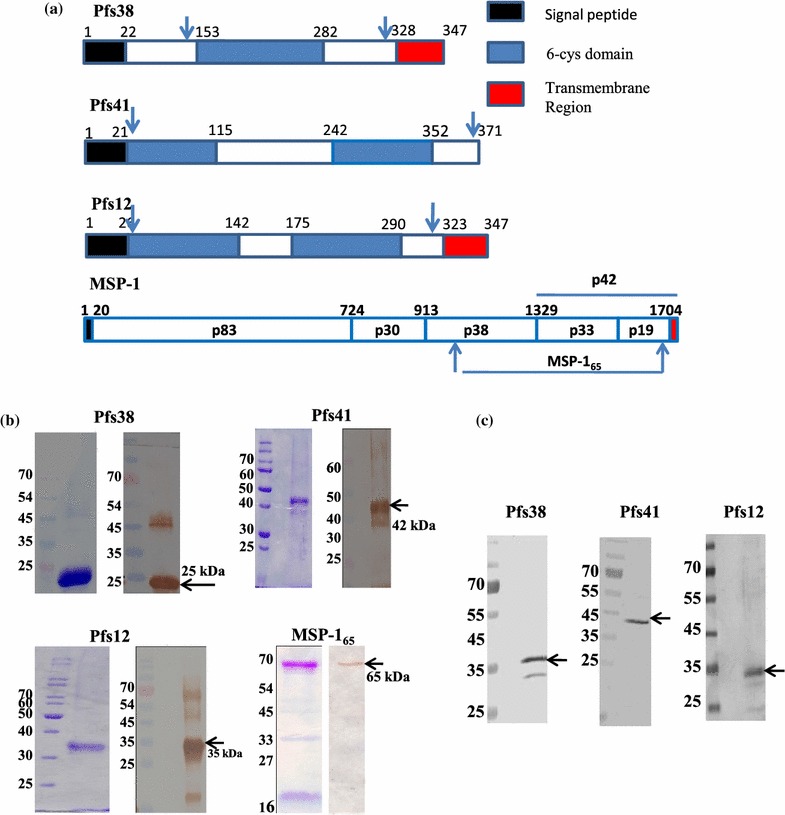
Expression of 6-Cys domain proteins: Pfs41, Pfs38, Pfs12 and PfMSP-1_65_. **a** Schematic showing the organization of the above-mentioned *Plasmodium* proteins. *Arrows mark* the regions that were expressed in *Escherichia coli.* **b** Coomassie stained SDS-PAGE and immunoblots showing the purified Pfs41, Pfs38, Pfs12 and PfMSP-1_65_. Immunoblots were performed using mouse monoclonal anti-his antibody. **c** Recognition of native Pfs41, Pfs38 and Pfs12 proteins in parasite lysate from asexual blood stages using their respective antibodies generated against recombinant proteins

Gene fragments encoding the 6-Cys proteins: Pfs38, Pfs41, Pfs12, and PfMSP-1_65_ were cloned in expression vector pET28b and recombinant proteins were expressed in *E. coli* (Fig. 1a). Recombinant 6-Cys proteins were purified to homogeneity under denaturing conditions and refolded by dilution into a refolding buffer containing glutathione, while PfMSP-1_65_ was produced in the same expression system under standard native conditions. The four recombinant proteins were purified to near homogeneity and were analysed in SDS PAGE as well as western blot using anti-His antibody (Fig. 1b). Pfs38, Pfs12 and Pfs41 showed a shift in migration pattern on SDS PAGE between reduced and non-reduced proteins while PfMSP-1_65_ being expressed as a soluble protein shows no difference in migration pattern (see Additional file 1). Specific antibodies were raised against these purified recombinant proteins in rabbit and mice. As demonstrated by western blot analysis of parasite-derived polypeptides, anti-Pfs38, anti-Pfs41 and anti-Pfs12 recognized bands of ~40, ~43 and ~35 sizes in the parasite lysates prepared from schizont stage (Fig. 1c). These antibodies also stained the merozoites and schizont stages, thereby indicating the expression of these 6-Cys proteins at asexual blood stages of *P. falciparum* (see Additional file 2A, B).

### Identification of Pfs38 protein complex at asexual blood stages

The possibility of a large Pfs38 protein complex was assessed by immunoprecipitation of parasite-derived polypeptides isolated from a highly synchronized culture of late schizonts with an anti-Pfs38 antibody. LC–MS/MS analysis of immunoprecipitated polypeptides identified Pfs38, GLURP, MSP-1, and SERA5, suggesting that these polypeptides exist in the form of a complex (Table 1). IgG purified from preimmune sera did not precipitate any of these proteins. To confirm the existence of such a complex, the same parasite extract was immunoprecipitated with an anti-PfMSP-1_65_ antibody followed by LC–MS/MS analysis. This analysis identified Pfs38, Pfs41 and SERA5 as interacting partners for MSP-1 (Table 2). These results were further corroborated in independent experiments where parasite extracts were immunoprecipitated with anti-Pfs12 and anti-Pfs41antibodies. These antibodies immunoprecipitated Pfs12, Pfs41, MSP-1, and SERA5, respectively (see Additional files 3 and 4). The full list of proteins identified in LC–MS/MS analysis for immunoprecipation with anti-Pfs38, anti-Pfs12, anti-Pfs41, anti-PfMSP-1_65_ and preimmune antibody is provided in Additional files 5, 6, 7, 8 and 9. Collectively, these results suggest that Pfs38 is a part of a large protein complex referred herewith as Pfs38 complex consisting of GLURP, MSP-1, SERA5, Pfs12, and Pfs41 proteins.

**Table 1:** Identification of malarial proteins immunoprecipitated using anti-Pfs38 antibody by LC/MS–MS analysis

Accession No.	Name of protein	Score	Sequence coverage (%)	Unique peptides	Sequences of peptides identified
PFE0395c	Pfs38	110	29.80	12	RYPNEEVKEEDR EEGNLYTSQFSVPPVVLTHR HSFSNSEIFER YPNEEVKEEDR YPNEEVKEEDRFNLNR KIPGcDFNADYK
PF10_0344	Glutamate rich protein (GLURP)	94	22.87	15	GQHEIVEVEEILPEDKNEK GQHEIVEVEEILPEDDKNEK KNEFSVVEEK SVSEPAEHVEIVSEK DGPVPSKPFEEIEK ETPVVDGPK VqHEIVEVEEILPEDK
PFI1475w	Merozoite surface protein 1 (MSP-1)	14	4.36	7	EAEIAETENTLENTK ALSYLEDYSLR YYNGESSPLK FPSSPPTTPPSPAK
PFB034c	Serine repeat antigen 5 (SERA-5)	0.00	3.61	3	TSPGLcLSK TNNAISFESNSGSLEK SYAFNPENYEK

**Table 2:** Identification of malarial proteins immunoprecipitated using anti-PfMSP-1_65_ antibody by LC/MS–MS analysis

Accession No.	Name of protein	Score	Sequence coverage (%)	Unique peptides	Sequences of peptides identified
PFI1475w	Merozoite surface protein 1 (MSP-1)	568	48	73	VPYPnGIVYPLPLTDIHNSLAADNDK INEIKNPPPANSGNTPNTLLDK TLSEVSIQTEDNYANLEK KLEALEDAVLTGYSLFQK LNSLNNPHNVLQNFSVFFNK YFLDVLESDLMQFK LLEVYNLTPEEENELK INEIKNPPPAnSGNTPNTLLDK EAEIAETENTLENTK
PFB0340c	Serine repeat antigen 5 (SERA-5)	70	12	10	NYAIGSDIPEKcDTLASNcFLSGNFNIEK ESNTALESAGTSNEVSER NYAIGSDIPEKcDTLASncFLSGNFNIEK cDTLASNcFLSGNFNIEK LPSnGTTGEQGSSTGTVR TNNAISFESNSGSLEK KYIDTQDVNKK EHNGTNLIESK ETPFTNILIHAYK LPSNGTTGEQGSSTGTVR
PFE0395c	Pfs38	15	12	4	YNVVSIETVLK HSFSNSEIFER SIGVNKHSFSNSEIFER MnMITQGDKYSIFSK
PFD0240c	Pfs41	7	9	2	GGNVSEAQADEYLNK SLNIPNDILNYDVYNSSNNR

### Protein–protein interaction analysis of Pfs38 with other merozoite surface proteins

**Fig. 2:**
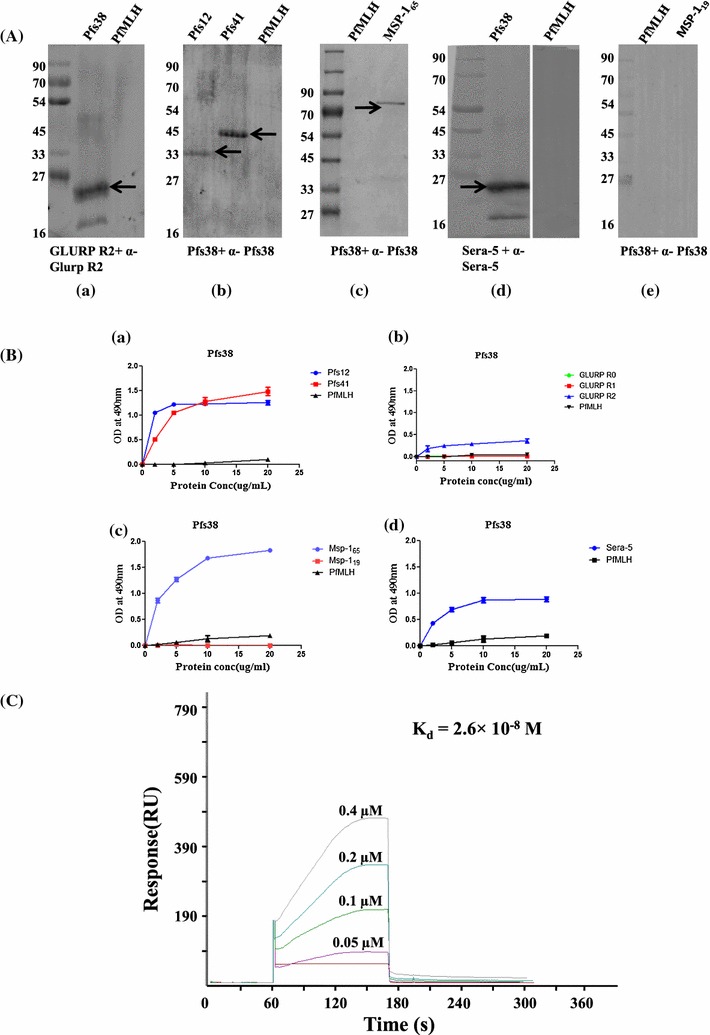
Existence of a Pfs38 protein complex at *Plasmodium* asexual blood stages. **A** Far western analysis showing interactions of recombinant Pfs38 with Pfs12 (*b*), Pfs41(*b*), GLURPR2 (*a*), PfMSP-1_65_(*c*), and SERA5 (*d*). *Plasmodium* PfMLH and PfMSP-1_19_ (*e*) did not show interaction with Pfs38. **B** ELISA binding analysis confirming the interactions of Pfs38 with Pfs12 (*a*), Pfs41 (*a*), GLURPR2 (*b*), PfMSP-1_65_ (*c*), and SERA5 (*d*) proteins, while *Plasmodium* PfMLH and PfMSP-1_19_ did not show binding to Pfs38. *Error bars* represent mean ± SEM of duplicate measurements. **C** SPR analysis showing an interaction between Pfs38 and GLURPR2

The direct interactions among the proteins of Pfs38 complex was examined by a number of biochemical tools. Far western analysis that detects the interaction between a membrane-bound prey protein and soluble bait proteins [36] was performed. Recombinant Pfs38 served as a prey protein while GLURP (R0, R1, R2), Pfs41, Pfs12, PfMSP-1_65_, PfMSP-1_19_ proteins and PfMLH protein [39] served as a bait in the two hybrid interaction analysis. As shown in Fig. 2a, Pfs38 interacted with the GLURPR2, SERA5, PfMSP-1_65_, Pfs12, and Pfs41 proteins, while no significant interaction was seen between Pfs38 and PfMSP-1_19_ or PfMLH proteins. Interactions among the proteins of Pfs38 complex were further confirmed by an ELISA-based binding assays in which recombinant Pfs38 was allowed to interact with different regions of GLURP (R0, R1, and R2), PfMSP-1_65_, PfMSP-1_19_, Pfs41, Pfs12, SERA5, and PfMLH. As shown in Fig. 2b, all protein–protein interactions seen in far western analysis were observed in the ELISA-based binding assay. Interestingly, no interaction was observed between Pfs38 and PfMSP-1_19_, suggesting that Pfs38 binds to a MSP-1 region upstream of the 19 kDa C-terminal region (Fig. 2b). A surface plasmon resonance analysis was next performed to assess the physical properties of the interaction between Pfs38 and GLURPR2. Recombinant GLURPR2 was immobilized on a CM5 sensor chip and increasing concentrations of recombinant Pfs38 were injected over the chip. Pfs38 bound to GLURPR2 in a dose-dependent manner with an equilibrium dissociation constant (K_d_) of 2.6 × 10^−8^ M (Fig. 2c). Together, these results confirmed the association between Pfs38 and other merozoite surface proteins.

### Co-localization and co-sedimentation analysis confirm an association of Pfs38 with other 6-cys proteins, SERA5 and PfMSP-1_65_ on the merozoite surface

**Fig. 3:**
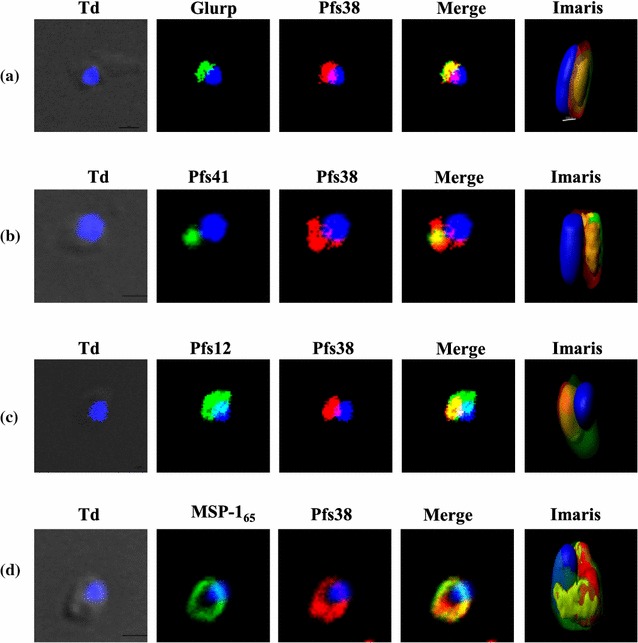
Expression and co-localization of proteins of Pfs38 complex on *Plasmodium* merozoites. Co-localization studies were performed by immunofluorescence assays. Merozoites were labelled with (**a**) mouse anti-Pfs38 and rabbit anti-GLURP or with (**b**) mouse anti-Pfs38 and rabbit anti-Pfs41 antibodies or with (**c**) mouse anti-Pfs38 and rabbit anti-Pfs12 antibodies or with (**d**) mouse anti-Pfs38 and rabbit anti-PfsMSP1_65_ antibodies. Partial co-localization was observed between Pfs38 and other proteins of 6-Cys complex with co-localization coefficient of 0.75, 0.61, 0.82, and 0.83 for **a**–**d**, respectively. *Td* represent *bright field images*

To know the localization of proteins of Pfs38 complex on the surface of *P. falciparum* merozoites, co-localization studies for these proteins were performed on intact merozoites by immunofluorescence staining using their specific antibodies. Pfs38 partially co-localized with GLURP, Pfs41, Pfs12, and MSP-1, advocating the co-existence of proteins of 6-Cys protein complex on the merozoite surface (Fig. 3a–d). These results were corroborated by co-sedimentation analysis of parasite-derived polypeptides. Western blotting and LC–MS/MS analysis of the glycerol gradient fractions revealed the presence of Pfs38, Pfs41, Pfs12, GLURP, MSP-1, and SERA5 in a single fraction, suggesting that these proteins exist in a complex on the parasite (see Additional files 10, 11).

### Pfs38 binds to human erythrocytes

**Fig. 4:**
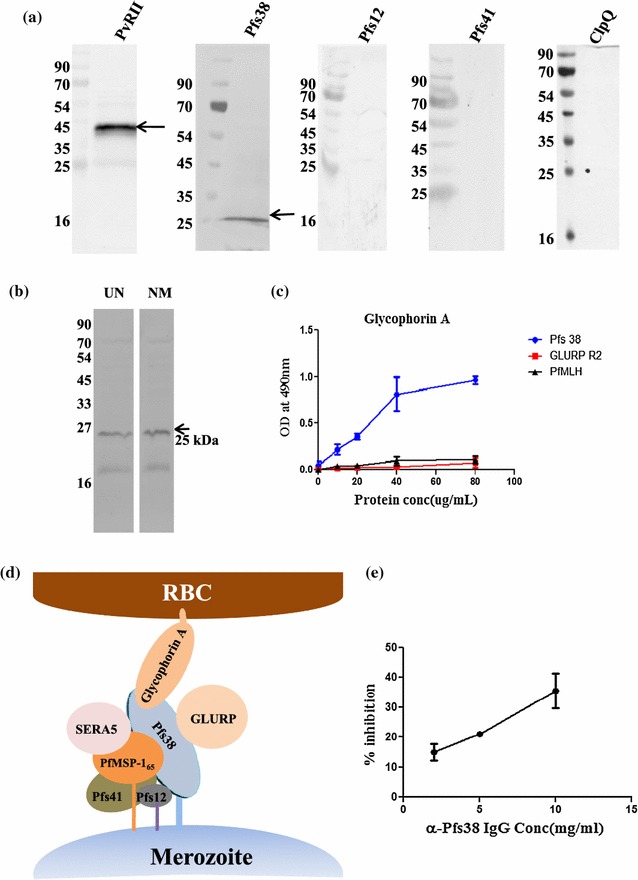
Pfs38 binds human erythrocytes via Glycophorin A and is required for invasion of red blood cells. **a** Recombinant Pfs38, Pfs12 and Pfs41 were incubated with uninfected human erythrocytes and bound proteins were eluted from the erythrocytes after centrifugation through oil. PvRII was used as a positive control for erythrocyte binding assay, while an internal protein ClpQ was used as a negative control for the assay; **b** Erythrocyte binding assay of recombinant Pfs38 with untreated (UN) and neuraminidase treated (NM) RBCs. **c** Glycophorin A is the erythrocyte receptor through which Pfs38 binds human erythrocytes. ELISA binding assay was performed to study the interaction between Glycophorin A and recombinant Pfs38. As a control, GLURPR2 and PfMLH protein were used. *Error bars* indicate mean ± SEM of duplicate measurements; **d** A model of Pfs38 protein complex based on protein–protein interactions and protein-erythrocyte interaction; **e** Invasion inhibition assay was performed on schizont stage using α-Pfs38 purified IgG at concentration of 2, 5 and 10 mg/mL and percent inhibition was calculated. *Bars* indicate mean ± SEM of duplicate measurements

The putative role of the Pfs38 complex in the invasion of *P. falciparum* merozoite into host RBC was also investigated. In vitro erythrocyte binding assays were performed with three recombinant 6-Cys proteins using intact human RBCs as described earlier [37]. *Plasmodium vivax* erythrocyte binding antigen region 2 (PvRII) [40] and ClpQ [41] were used as positive and negative controls, respectively. Pfs38 efficiently bound to human erythrocytes and the binding was found to be neuraminidase resistant. In contrast, neither Pfs12 or Pfs41 showed appreciable binding to human erythrocytes (Fig. 4a, b). No erythrocyte binding activity has been reported for GLURP and SERA5 so far. An earlier study using a cross-linking assay with synthetic peptides derived from Pfs38, Pfs41 and Pfs12 and RBC membrane proteins indicated that these proteins might be binding to members of glycophorin family present on the RBC membrane [42]. Although the binding of Pfs38 to human RBC was neuraminidase resistant, an ELISA-based interaction was still carried out between Pfs38 and glycophorin A protein.

Recombinant Pfs38 showed a dose-dependent interaction with glycophorin A (Fig. 4c), while GLURP R2 from the same complex and PfMLH (non-specific protein) did not show interaction to glycophorin A. These results are in line with a recent finding where MSP-1 amino terminus region (Msp-1_83_) was found to bind with glycophorin A of human RBC even in the presence of neuraminidase treatment [43]. Furthermore anti-Pfs38 antibodies could inhibit the interaction between Pfs38 and glycophorin A by up to 70% at 2 mg/mL (see Additional file 12). Together, these results show that Pfs38 protein of the Pfs38 protein complex binds to human erythrocyte via glycophorin A. Based on the results of interactions studies among the proteins of Pfs38 protein complex and interaction of Pfs38 with RBC surface, a model is proposed for the organization of Pfs38 protein complex (Fig. 4d) showing that Pfs12 and Pfs38 proteins are anchored to merozoite membrane by GPI domains and Pfs41 interacts with these proteins to stabilize them. Pfs38 binds RBC surface via glycophorin A, while PfMSP-1_65_, GLURP and SERA5 bind to the axis generated by Pfs41, Pfs12 and Pfs38.

### Invasion inhibition assay (GIA)

The inhibitory potential of anti-Pfs38 antibodies on the invasion of *Plasmodium* merozoites into RBCs were evaluated. An invasion inhibition assay was performed on *P. falciparum* 3D7 using the purified anti-Pfs38 IgG at different concentrations. IgG purified from pre-immune sera served as a negative control. Anti-Pfs38 IgG showed an inhibition of approximately 30–40% at a concentration of 10 mg/mL (Fig. 4e).

### Humoral immune responses to the proteins associated with Pfs38 protein complex

**Fig. 5:**
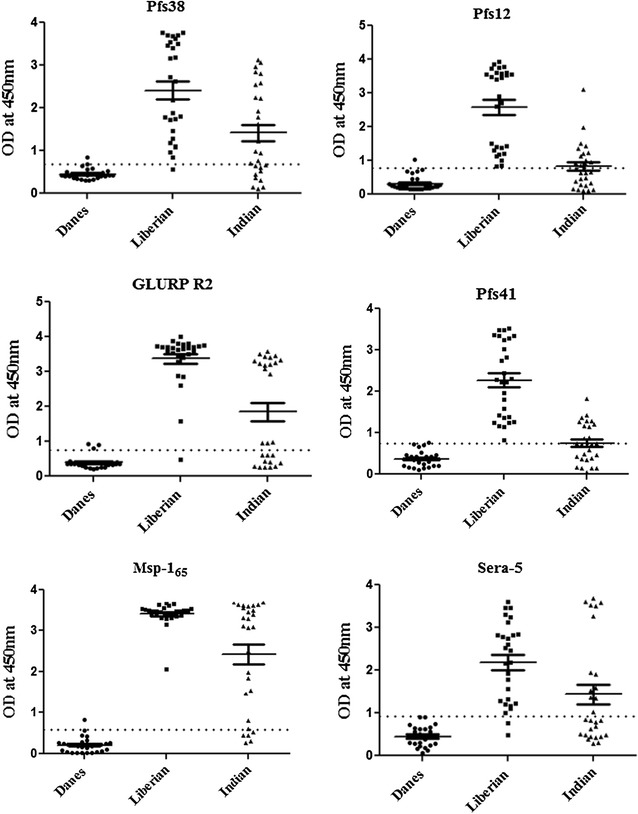
Naturally-acquired humoral IgG immune responses to proteins of Pfs38 complex. Human IgG antibodies against proteins of 6-Cys complex were detected by ELISA in sera from naturally infected patients from Liberia and India. Sera from Denmark were used as a negative control. The *dotted line* indicates positivity thresholds determined from the mean reactivities +2 SD of 28 sera samples from Danish non-immune volunteers. African and Indian sera with OD above the positive thresholds are considered seropositive for each of the antigen. *Bars* represent mean ± SEM for 28 sera samples

The immunogenicity of the members of Pfs38 complex were evaluated during natural infections using plasma from Africa and India by ELISA. MSP-1_65_, GLURPR2, Pfs38, Pfs12, Pfs41, and SERA5 proteins were frequently recognized by sera from Liberia in Africa with seropositivity rates of 100, 96, 89, 89, 96, and 89%, respectively (see Fig. 5; Additional file 13). Interestingly, these antigens were also recognized by sera from India with seropositivity rates of 80, 60, 60, 42, 40, and 46%, respectively (see Fig. 5; Additional file 13). The lower seropositivity rates observed among Indian samples may be related to a lower transmission intensity in this area compared with that of Liberia. The seropositivity of three refolded 6-cys proteins, i.e. Pfs38, Pfs12 and Pfs41 as well as PfMsp-1_65_, were significantly reduced when these proteins were denatured with 8 M urea, thereby confirming their proper folding (see Additional file 14).

Irrespective of the differential reactivity, the relative high seroprevalence rates in both areas indicate that the 6-Cys proteins: Pfs38, Pfs12, Pfs41, and MSP-1 as well as SERA proteins used in the present study, are good immunogen and these proteins have adopted their natural folds. Taken together, these results indicate that proteins of the Pfs38 complex are expressed during the natural infection in geographical distinct parasite populations.

## Discussion

An effective blood-stage vaccine to prevent the prevalence of malaria or ameliorate the severity of disease is still a distant prospect because of major gaps in the knowledge of *Plasmodium* biology, especially in the understanding of molecular architecture of merozoite surface as well as the critical events involved in the invasion of RBCs. Among the many antigens that coat merozoite surface, 6-Cys proteins are of significant interest as they are expressed at multiple stages of *P. falciparum* life cycle and have a characteristic arrangement of cysteine residues [18, 29, 44–46]. Although 6-Cys domain proteins were identified more than 20 years ago, their functions at asexual blood stages are yet to be defined.

To get insights into the role of three 6-Cys proteins: Pfs41, Pfs38 and Pfs12, at asexual blood stages, particularly in the invasion process, these proteins were expressed and purified in *E. coli* expression systems and specific antibodies to these proteins were raised. These antibodies were specific as they recognized specific bands in asexual blood stages of *P. falciparum* lysates and stained the merozoite surface. It has been shown that Pfs12 and Pfs41 exist as heterodimer on the *P. falciparum* merozoite surface [20]. To know whether these proteins are part of a large network, the *P. falciparum* schizont stage parasite lysate was immunoprecipitated using either anti-Pfs38 or anti-Pfs41 or anti-Pfs12 antibody and the immunoprecipitated samples were analysed by LC–MS/MS. A similar approach has been adopted by us and others to identify protein complexes in *Plasmodium* and other organisms [47]. These analyses provided evidence for the association of three 6-cys proteins (Pfs38, Pfs12 and Pfs41) with other merozoite surface proteins: GLURP, PfMSP-1 and SERA-5 on the merozoite surface. Careful analysis of the peptides generated in MS/MS analysis showed that a region upstream of the PfMSP-1_19_ is associated with the complex. Overall, these results suggested the existence of a large Pfs38 protein complex comprising of GLURP, SERA5, PfMSP-1, Pfs41, and Pfs12 proteins.

In vitro protein–protein interaction tools, such as ELISA based binding assays and Far western blot analysis between the six proteins of Pfs38 protein complex and co-localization studies, provided additional evidence for the existence of such a complex. The interaction between Pfs38 and GLURPR2 was further quantified by surface plasmon resonance (SPR) analysis in the present study. Although a couple of previous studies have shown an interaction between Pfs12/Pvs12 and Pfs41/Pvs41 [20, 40], other interactions between the components of Pfs38 complex have never been reported. Among the merozoite proteins identified in Pfs38 complex, Pfs12 and Pfs38 anchors to the merozoite membrane via GPI motif similar to several other merozoite surface proteins, while Pfs41, SERA5 and GLURP lack GPI-anchoring motifs and they interact with Pfs12 and Pfs38 via non-covalent interactions [16]. Although MSP-1 has a GPI anchoring motif, nonetheless this protein undergoes processing and region(s) of MSP-1 that interacts with this complex are distant from PfMSP-1_19_, a region anchored to the merozoite membrane [48].

Gene knock-out studies have shown that neither Pfs12 nor Pfs41 are essential for the malaria parasite growth or invasion and antibodies to these antigens do not significantly block erythrocyte invasion in vitro [20]. These proteins also did not show binding to human erythrocyte in an in vitro assay [20]. As the structural and biochemical characteristics of 6-Cys proteins predict their role(s) in the process of recognition and adhesion of host cells [26, 29], the possibility of Pfs38, Pfs41 and Pfs12 interaction with human RBCs was explored. As shown previously, Pfs41 and Pfs12 did not show binding to human erythrocytes, however, Pfs38 bound to RBCs via glycophorin A and anti-Pfs38 antibodies showed a moderate invasion (~40%) inhibition activity. These results are in line with a previous study where anti-Pfs38 antibody generated against a plant-produced recombinant Pfs38 showed inhibition of ≥60% [49]. Although a moderate level of invasion inhibition was observed with anti-Pfs38 antibody, it is possible that for greater protection antibodies to other components of the complex may also be required. This is evident in an earlier report that showed better protection against a *Plasmodium yoelii* challenge in mice immunized with PfAMA-1-RON2 complex in comparison to the mice immunized with each antigen alone [50]. Together these studies advocate the development of a sub-unit malaria vaccine based on the candidates that are present in complexes with one or more binding partners instead of the individual component.

To explore whether the recombinant proteins used in interaction analysis were properly folded with intact conformational epitopes and are targets for naturally acquired humoral immunity, these six antigens of Pfs38 protein complex were screened against plasma from 28 Liberian and 28 *P. falciparum*-infected Indian patients. All the six antigens: Pfs38, Pfs12, Pfs41, PfMSP-1_65_, GLURPR2, and SERA5 showed at least a two-fold change in IgG reactivity between the naïve sera and *P. falciparum*-exposed plasma in two geographically distinct populations. However, antibody responses among six antigens varied. GLURP R2 and PfMSP-1_65_ showed significantly high antibodies than members of the 6-Cys family. Similar high responses have been observed against GLURPR2 in a number of previous studies [33, 51, 52]. Differences in the humoral responses against these antigens between the Liberian and Indian sera were observed. The difference in the reactivity observed between Indian and Liberian sera may be because Liberia is a malaria-endemic region, while Indian isolates were from non-endemic areas. Interestingly denaturing four of these antigens by urea treatment significantly reduced their reactivity with Liberian sera thereby further confirming the folding of recombinant proteins used in the present study.

## Conclusions

In summary, the existence of a large Pfs38 complex on *P. falciparum* merozoites consisting of three 6-Cys family members: Pfs38, Pfs41 and Pfs12 that associate with GLURP, PfMSP-1_65_ and SERA5 was revealed. Pfs38 protein of this large Pfs38 complex binds to RBC via glycophorin A. Two proteins of this complex: GLURP and PfMSP-1_65_ showed high seroreactivity to sera from *P. falciparum*-infected patients with a seroprevalence of >90% from two geographically distinct populations. Although broader epidemiological investigations and in vivo challenge studies are required, the results of the present study point towards developing malaria sub-unit vaccines based on merozoite surface protein complexes.

## Additional files

**Additional file 1.** A western Blot of reduced(R) and non-reduced(NR) Pfs38, Pfs12, Pfs41 and PfMSP-1_65_ using anti-His antibody.

**Additional file 2.** Immunolocalization of Pfs38, Pfs41and Pfs12 in (A) schizont and (B) merozoite stage of the parasite using antibodies raised against these proteins.

**Additional file 3.** Identification of malarial proteins immunoprecipitated using anti-Pfs12 antibodies by LC-MS/MS.

**Additional file 4.** Identification of malarial proteins immunoprecipitated using anti-Pfs41 antibodies by LC-MS/MS.

**Additional file 5.** Full list of proteins identified in LC-MS/MS analysis for immunoprecipitation with α-Pfs38 antibody.

**Additional file 6.** Full list of proteins identified in LC-MS/MS analysis for immunoprecipitation with α-Pfs12 antibody.

**Additional file 7.** Full list of proteins identified in LC-MS/MS analysis for immunoprecipitation with α-Pfs41 antibody.

**Additional file 8.** Full list of proteins identified in LC-MS/MS analysis for immunoprecipitation with α-PfMSP-1_65_ antibody.

**Additional file 9.** Full list of proteins identified in LC-MS/MS analysis for immunoprecipitation with preimmune IgG.

**Additional file 10.** Evidence for the existence of Pfs38 complex by sedimentation analysis. Glycerol gradient fractionation of *Plasmodium* schizont extract using 5 to 45% glycerol gradient and immunoblotting of glycerol gradient fractions using anti-Pfs38, anti-Pfs41, anti-Pfs12, anti-GLURP, anti-PfMSP-1_65_ and anti-SERA5 antibodies. Note the co-sedimentation of Pfs41, Pfs38, Pfs12, GLURP, PfMSP-1_65_ and Sera-5 in fraction 5.

**Additional file 11.** Identification of malarial proteins in fraction 5 of glycerol density gradient by LC-MS/MS analysis.

**Additional file 12.** Inhibition of Pfs38 glycophorin A interaction by anti-Pfs38 antibodies.

**Additional file 13.** Seropositivity rates of members of Pfs38 complex in Indian and Liberian sera as determined by ELISA.

**Additional file 14.** Comparison of seropositivity of untreated and 8M urea denatured proteins for Pfs38, Pfs41, Pfs12 and PfMSP-1_65._
