# Supplementary figures and images for: A novel Pfs38 protein complex on the surface of Plasmodium falciparum blood-stage merozoites
Source: Malar J. 2017 Feb 16;16:79. doi: 10.1186/s12936-017-1716-0 (PMC5312596; doi:10.1186/s12936-017-1716-0)

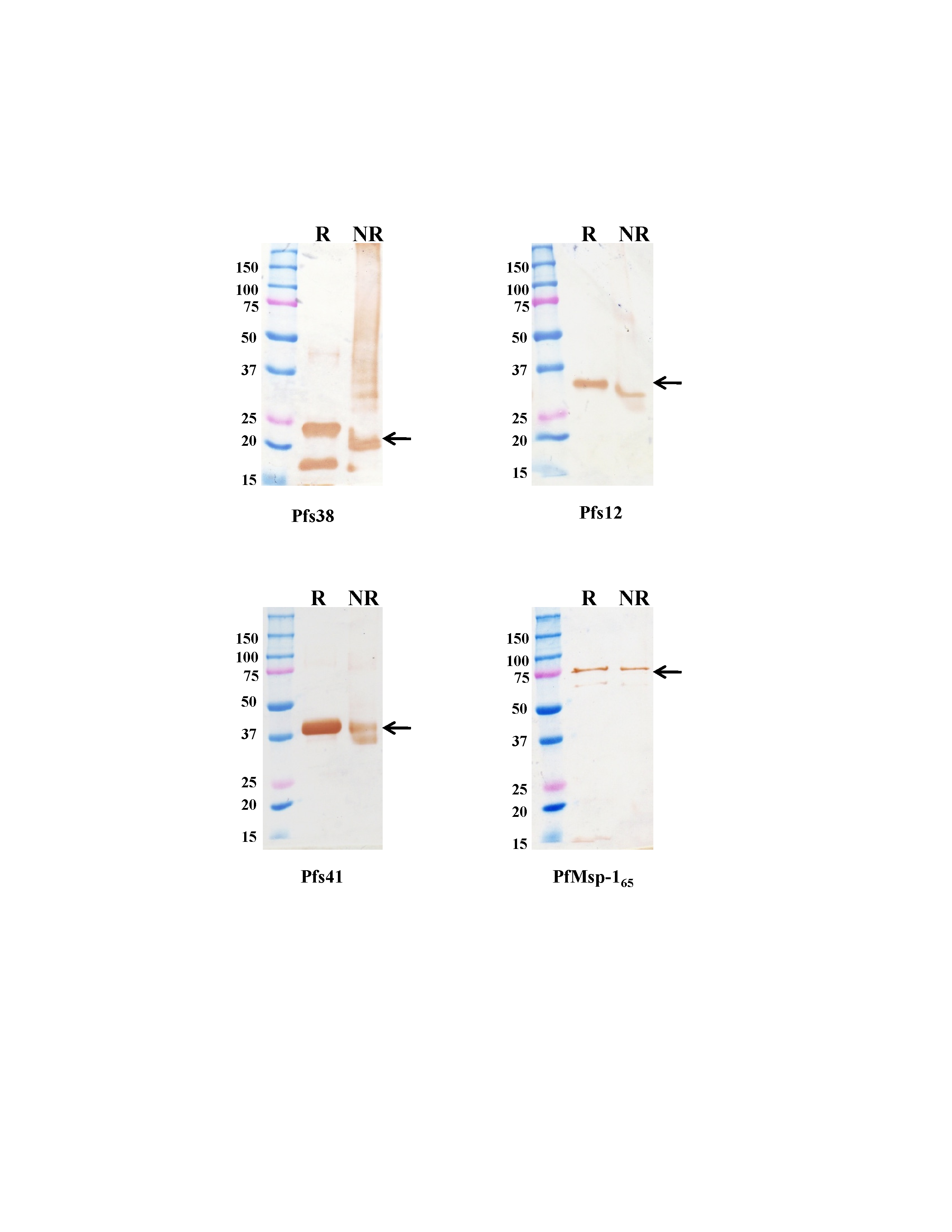

Supplement: Supplementary file 1 — Additional file 1. A western Blot of reduced(R) and non-reduced(NR) Pfs38, Pfs12, Pfs41 and PfMSP-165 using anti-His antibody. [file 12936_2017_1716_MOESM1_ESM.tif]

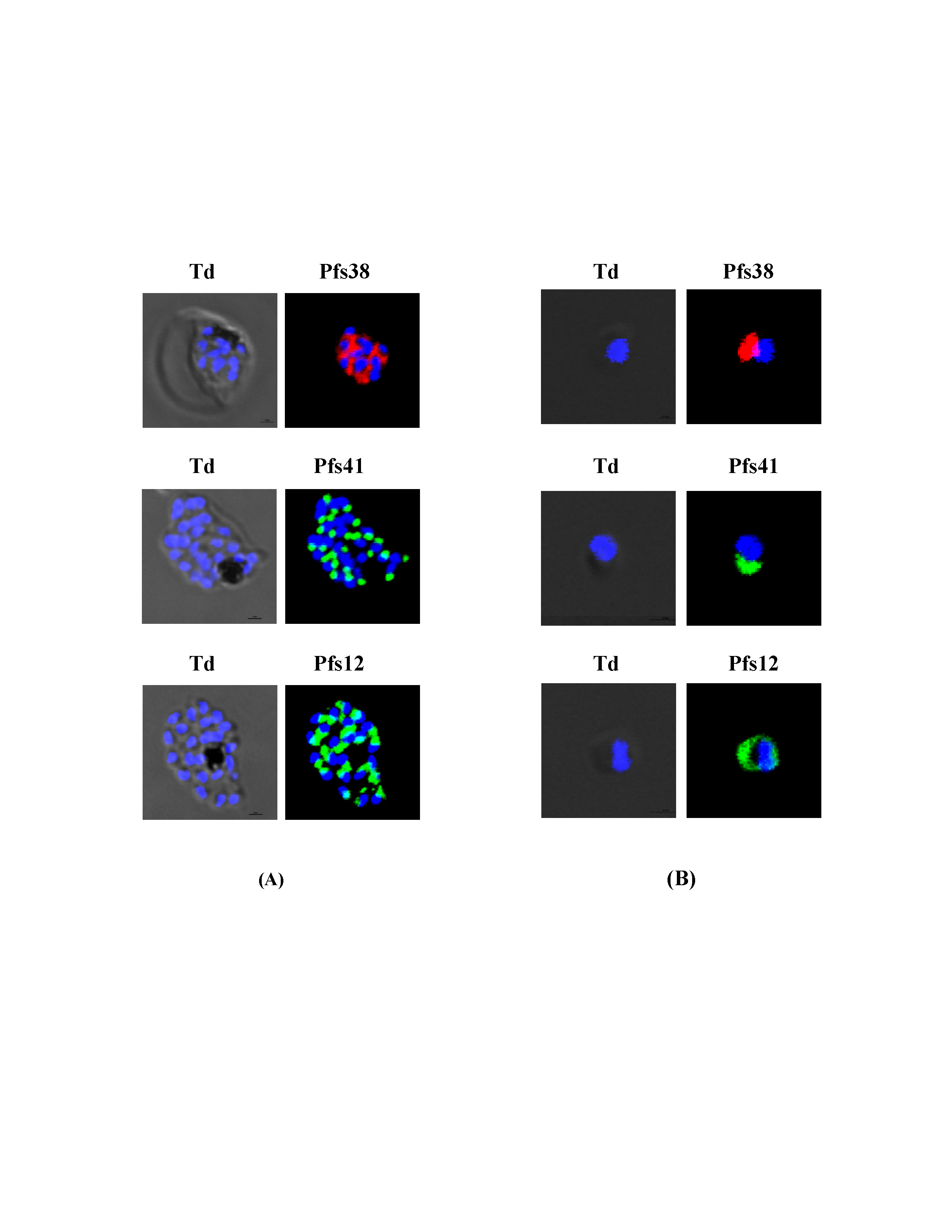

Supplement: Supplementary file 2 — Additional file 2. Immunolocalization of Pfs38, Pfs41and Pfs12 in (A) schizont and (B) merozoite stage of the parasite using antibodies raised against these proteins. [file 12936_2017_1716_MOESM2_ESM.tif]

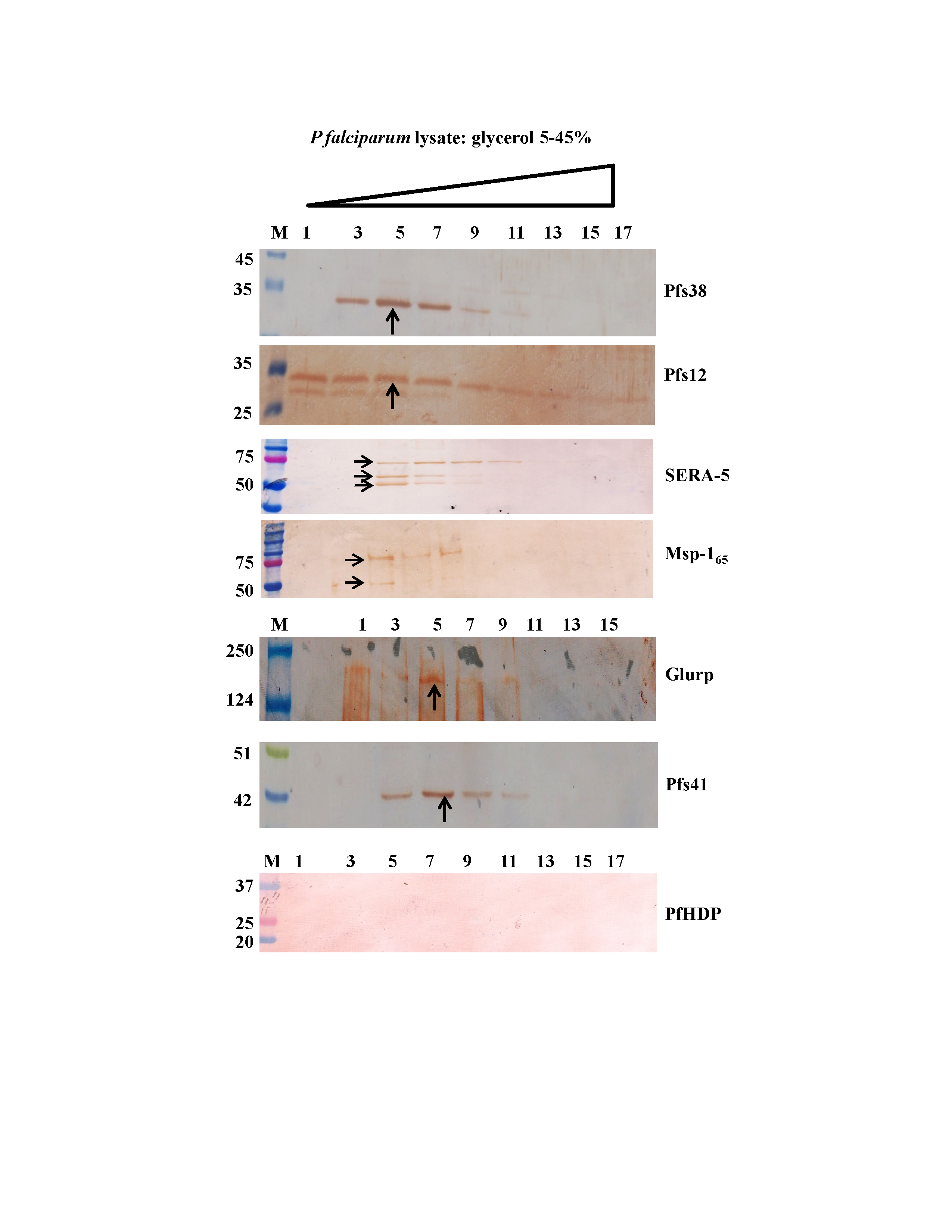

Supplement: Supplementary file 10 — Additional file 10. Evidence for the existence of Pfs38 complex by sedimentation analysis. Glycerol gradient fractionation of Plasmodium schizont extract using 5 to 45% glycerol gradient and immunoblotting of glycerol gradient fractions using anti-Pfs38, anti-Pfs41, anti-Pfs12, anti-GLURP, anti-PfMSP-165 and anti-SERA5 antibodies. Note the co-sedimentation of Pfs41, Pfs38, Pfs12, GLURP, PfMSP-165 and Sera-5 in fraction 5. [file 12936_2017_1716_MOESM10_ESM.tif]

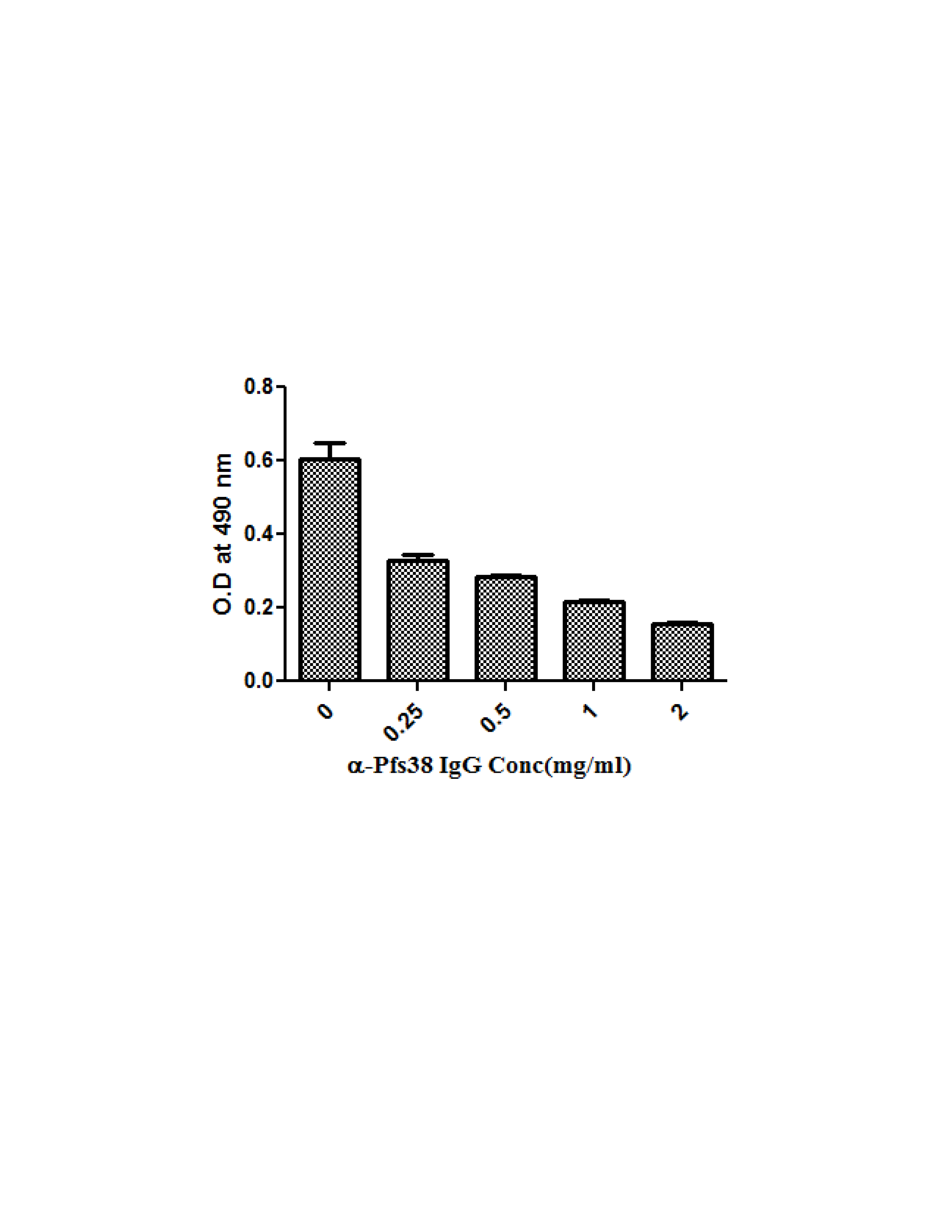

Supplement: Supplementary file 12 — Additional file 12. Inhibition of Pfs38 glycophorin A interaction by anti-Pfs38 antibodies. [file 12936_2017_1716_MOESM12_ESM.tif]

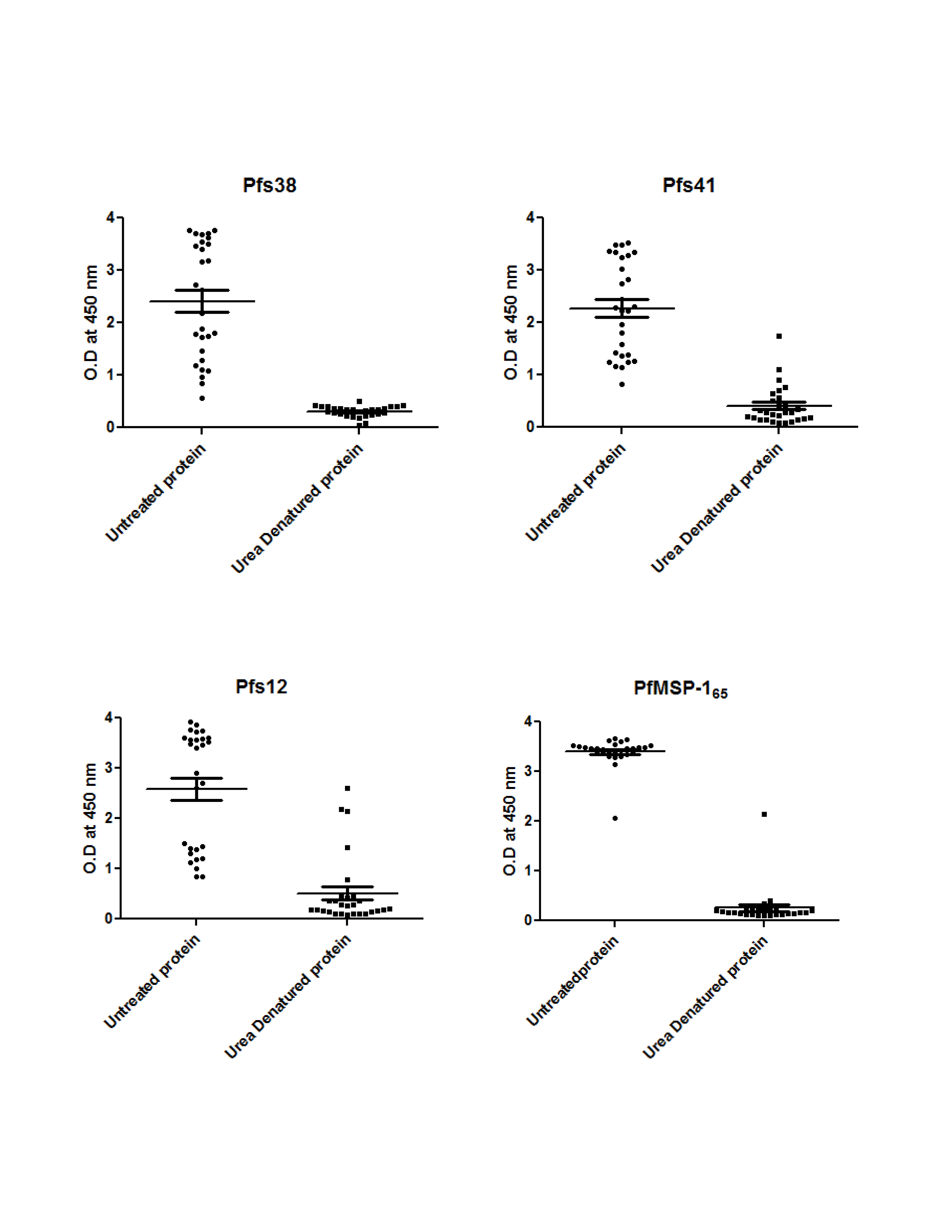

Supplement: Supplementary file 14 — Additional file 14. Comparison of seropositivity of untreated and 8M urea denatured proteins for Pfs38, Pfs41, Pfs12 and PfMSP-165. [file 12936_2017_1716_MOESM14_ESM.tif]
