# Supplementary material for: A novel Pfs38 protein complex on the surface of Plasmodium falciparum blood-stage merozoites
Source: Malar J. 2017 Feb 16;16:79. doi: 10.1186/s12936-017-1716-0 (PMC5312596; doi:10.1186/s12936-017-1716-0)
Supplement: Supplementary file 3 — Additional file 3. Identification of malarial proteins immunoprecipitated using anti-Pfs12 antibodies by LC-MS/MS. [file 12936_2017_1716_MOESM3_ESM.docx]

| **Accession No** | **Name of the Protein** | **Score** | **Sequence Coverage (%)** | **Unique peptides** | **Sequences of Peptides Identified** |
| --- | --- | --- | --- | --- | --- |
| PFF0615c | Pfs12 | 17.34 | 20.4 | 5 | GcDFTTSESTIFSK  SSNNQQDIVcTVK  GIVEIIIPSLNEK  LNYNLYPPTcFEEVYASR  LEFHPNQQTSVTK |
| PFD0240c | Pfs41 | 44 | 35 | 9 | GGNVSEAQADEYLNK  EVScEIDANPSDDITFIcPNK  TNTPFYcFcNLDTVTIQK  SLNIPNDILNYDVYNSSNNR  IDSLcFHTVNISK  NYFSHPISVAGK  TLIPGYASYTNK  IQGKPGELVGFK |
| PFI1475w | Merozoite surface protein 1  (MSP-1) | 71 | 10 | 14 | KLEALEDAVLTGYSLFQK  TLSEVSIQTEDNYANLEK  EAEIAETENTLENTK  LEALEDAVLTGYSLFQK  LLEVYNLTPEEENELK  EAEIAETEnTLEnTK  MVLNEEEITTK  TTIANINELIEGSK  NNDTYFNDDIK  SYENFLPEAK  ALSYLEDYSLR |
| PFB0340c | Serine repeat antigen 5 (SERA-5) | 3.2 | 2 | 1 | LPSNGTTGEQGSSTGTVR |

Identification of malarial proteins immunoprecipitated using anti-Pfs 12 antibody by LC/MS-MS analysis
