# Supplementary material for: A novel Pfs38 protein complex on the surface of Plasmodium falciparum blood-stage merozoites
Source: Malar J. 2017 Feb 16;16:79. doi: 10.1186/s12936-017-1716-0 (PMC5312596; doi:10.1186/s12936-017-1716-0)
Supplement: Supplementary file 4 — Additional file 4. Identification of malarial proteins immunoprecipitated using anti-Pfs41 antibodies by LC-MS/MS. [file 12936_2017_1716_MOESM4_ESM.docx]

| **Accession No** | **Name of the Protein** | **Score** | **Sequence Coverage (%)** | **Unique peptides** | **Sequences of Peptides Identified** |
| --- | --- | --- | --- | --- | --- |
| PFD0240c | Pfs41 | 55 | 41 | 10 | IDSLCFHTVNISK  GGNVSEAQADEYLNK  IPHFVNEQYTIQCK  TNTPFYCFCNLDTVTIQK  YLLSGEKEVSCEIDANPSDDITFICPNK  SLNIPNDILNYDVYNSSNNR  FFNDQADNTTK  NDIVVKDEVTNK  IQGKPGELVGFK |
| PFF0615c | Pfs12 | 3.02 | 3 | 1 | GIVEIIIPSLNEK |
| PFI1475w | Merozoite surface protein 1  (MSP-1) | 167 | 29 | 41 | INDCNVEKDEAHVK  TTIANINELIEGSKK  GLTHSANGSLEVSDIVK  LKDTLQLSFDLYNK  QLEEAHNLISVLEK  YKDDLESIKK |
| PFB0340c | Serine repeat antigen 5 (SERA-5) | 24 | 9 | 6 | ETPFTNILIHAYK  ESNTALESAGTSNEVSER  TNNAISFESNSGSLEK  CDTLASNCFLSGNFNIEK  LTESIDNILVK  NSWGPYWGDEGYFK |

Identification of malarial proteins immunoprecipitated using anti-Pfs 41 antibody by LC/MS-MS analysis
