# Supplementary material for: A novel Pfs38 protein complex on the surface of Plasmodium falciparum blood-stage merozoites
Source: Malar J. 2017 Feb 16;16:79. doi: 10.1186/s12936-017-1716-0 (PMC5312596; doi:10.1186/s12936-017-1716-0)
Supplement: Supplementary file 11 — Additional file 11. Identification of malarial proteins in fraction 5 of glycerol density gradient by LC-MS/MS analysis. [file 12936_2017_1716_MOESM11_ESM.docx]

| **Accession No** | **Name of the Protein** | **Score** | **Sequence Coverage (%)** | **Unique peptides** | **Sequences of Peptides Identified** |
| --- | --- | --- | --- | --- | --- |
| **PFE0395c** | Pfs38 | 10.03 | 12.32 | 3 | FVPLNLVPGDVVEYScPYSLNNDIR  YNVVSIETVLK  VcDVYPK |
| **PF10_0344** | Glutamate rich protein  (GLURP) | 3.48 | 0.97 | 1 | EKENVSEVVEEK |
| **PFI1475w** | Merozoite surface protein 1  (MSP-1) | 10.28 | 1.05 | 1 | FNIDSLFTDPLELEYYLR |
| **PFB0340c** | Serine repeat antigen 5 (SERA-5) | 113.14 | 30.89 | 26 | LKDENNcISNLQVEDQGNcDTSWIFASK  NYAIGSDIPEKcDTLASncFLSGNFNIEK  ESNTALESAGTSNEVSER  KVQNLcGDDTADHAVnIVGYGNYVNSEGEK  LLIYHSEENINTLK  LPSnGTTGEQGSSTGTVR  SYAFNPENYEK  GLVLPELNYDLEYFNEHLYNDK  cDTLASNcFLSGNFnIEK |
| **PFD0240c** | Pfs41 | 1.69 | 3.17 | 1 | TLIPGYASYTNK |

Identification of Malarial proteins in fractions of Glycerol density gradient centrifugation by LC-MS/MS analysis
