# Supplementary material for: A novel Pfs38 protein complex on the surface of Plasmodium falciparum blood-stage merozoites
Source: Malar J. 2017 Feb 16;16:79. doi: 10.1186/s12936-017-1716-0 (PMC5312596; doi:10.1186/s12936-017-1716-0)
Supplement: Supplementary file 13 — Additional file 13. Seropositivity rates of members of Pfs38 complex in Indian and Liberian sera as determined by ELISA. [file 12936_2017_1716_MOESM13_ESM.docx]

| **Antigens** | **Seropositivity in Liberian sera(%)** | **Seropositivity in Indian sera(%)** |
| --- | --- | --- |
| Pfs 38 | 89 | 60 |
| Pfs 12 | 89 | 42 |
| Pfs 41 | 96 | 40 |
| MSP-1_65_ | 100 | 80 |
| SERA-5 | 89 | 46 |
| GLURP R2 | 96 | 60 |

Seropositivity rates of members of 6-cys complex in Indian and Liberian sera as determined by ELISA
